# Energy compensation after sprint- and high-intensity interval training

**DOI:** 10.1371/journal.pone.0189590

**Published:** 2017-12-15

**Authors:** Matthew M. Schubert, Elyse Palumbo, Rebekah F. Seay, Katie K. Spain, Holly E. Clarke

**Affiliations:** 1 Department of Kinesiology, California State University–San Marcos, San Marcos, CA, United States of America; 2 Department of Kinesiology, Auburn University at Montgomery, Montgomery, AL, United States of America; 3 Department of Kinesiology and Health Promotion, University of Kentucky, Lexington, KY, United States of America; 4 Edward Via College of Osteopathic Medicine, Auburn Campus, Auburn, AL, United States of America; 5 Department of Nutrition, Food, and Exercise Sciences, Florida State University, Tallahassee, FL, United States of America; University of Alabama at Birmingham, UNITED STATES

## Abstract

**Background:**

Many individuals lose less weight than expected in response to exercise interventions when considering the increased energy expenditure of exercise (ExEE). This is due to energy compensation in response to ExEE, which may include increases in energy intake (EI) and decreases in non-exercise physical activity (NEPA). We examined the degree of energy compensation in healthy young men and women in response to interval training.

**Methods:**

Data were examined from a prior study in which 24 participants (mean age, BMI, & VO_2_max = 28 yrs, 27.7 kg•m^-2^, and 32 mL∙kg^-1^∙min^-1^) completed either 4 weeks of sprint-interval training or high-intensity interval training. Energy compensation was calculated from changes in body composition (air displacement plethysmography) and exercise energy expenditure was calculated from mean heart rate based on the heart rate-VO_2_ relationship. Differences between high (≥ 100%) and low (< 100%) levels of energy compensation were assessed. Linear regressions were utilized to determine associations between energy compensation and ΔVO_2_max, ΔEI, ΔNEPA, and Δresting metabolic rate.

**Results:**

Very large individual differences in energy compensation were noted. In comparison to individuals with low levels of compensation, individuals with high levels of energy compensation gained fat mass, lost fat-free mass, and had lower change scores for VO_2_max and NEPA. Linear regression results indicated that lower levels of energy compensation were associated with increases in ΔVO_2_max (p < 0.001) and ΔNEPA (p < 0.001).

**Conclusions:**

Considerable variation exists in response to short-term, low dose interval training. In agreement with prior work, increases in ΔVO_2_max and ΔNEPA were associated with lower energy compensation. Future studies should focus on identifying if a dose-response relationship for energy compensation exists in response to interval training, and what underlying mechanisms and participant traits contribute to the large variation between individuals.

## Background

Individual responses to an exercise intervention are highly variable, but are often hidden by the reporting of typical descriptive statistics (i.e. mean and standard deviation) [1–4]. In response to the same dose of exercise, some individuals may lose large amounts of weight while others may not, or even gain weight [4–6]. Maximal aerobic power (VO_2_max) [7, 8], resting metabolic rate (RMR) [4, 9], and body composition [4, 7] also exhibit high levels of variation in response to exercise training.

In theory, the undertaking of an exercise intervention (i.e. increasing energy expenditure) should shift energy balance into an energy deficit, leading to considerable weight loss [3, 10]. However, a number of highly controlled studies have observed that even in response to supervised and carefully administered exercise, considerable variability in weight changes occurred [5–7, 10–13]. This has led to researchers becoming interested in the causes of energy compensation in response to an exercise intervention. Among individuals who compensated in response to an exercise intervention, some authors have reported increases in energy intake [4, 12] and perceived hunger [12], lower levels of non-exercise physical activity (NEPA) [4], and smaller changes in VO_2_max [13]. Determining the reason(s) for differing patterns of individual response is of interest to the exercise physiologist in order to develop the optimum individual exercise prescription that would result in minimal levels of compensation.

Given the present physical activity guidelines of 150 min∙wk^-1^ of moderate exercise to accumulate health benefits, or up to 300 min∙wk^-1^ to promote weight loss, novel strategies to find the most time-efficient exercise intervention have been explored. In particular, recent research has focused on sprint- and high-intensity interval training (SIT and HIIT, respectively) [14]. Interval training is characterized by brief, intense bouts separated by recovery periods in a work to rest ratio of ≥ 1:1 [14]. Interval exercise is equally effective, or superior, to moderate-intensity continuous training for improving many health-related variables, including: increased VO_2_max and altered substrate use during exercise [15, 16], RMR [17], upregulation of skeletal muscle proteins and markers of mitochondrial function related to oxidative phosphorylation capacity [18, 19], reduced insulin resistance [20], and improved body composition [18, 21]. However, the compensatory responses to interval training have not previously been examined.

Therefore, the aim of the present paper was to examine individual differences in energy compensation in response to short-term interval training. Based on prior reports examining compensation to aerobic exercise [6, 10, 13], we hypothesized that the degree of energy compensation would be associated with changes in energy intake, physical activity, and/or VO_2_max.

## Methods

The present paper is a secondary analysis of a study previously published [17]. That study sought to examine if 4 weeks of SIT or HIIT influenced resting metabolic rate (RMR), resting substrate oxidation, body composition, non-exercise physical activity (NEPA), energy intake (EI), exercise metabolism, and VO_2_max.

### Participants

As previously described [17], participants were 24 moderately active (defined as > 120 minutes of moderate/vigorous activity per week by self-report during the previous 6 months) men and women (58% female) between the ages of 18–50; non-smokers; not currently taking any medication or supplements known to effect metabolism or blood pressure; and (for women) were eumenorrheic or on birth control, and not planning on becoming pregnant in the following 3 months. Participants typically completed recreational aerobic-type exercise (walking, jogging, cycling) as well as resistance training 3–5 d∙wk^-1^. They were asked to maintain their dietary and physical activity habits during the study period. This study was approved by the Auburn University at Montgomery Institutional Review Board for Human Subjects Research and adhered to the guidelines laid out in the Declaration of Helsinki.

### Exercise interventions

The full details of the exercise interventions have been previously published [17]. Briefly, participants were randomized to SIT or HIIT for 3 sessions∙wk^-1^ for 4 weeks. The SIT group completed training as previously described by Gillen [22], with modifications. Training the first week of the study consisted of a 2-minute warm-up at 10% peak power output (PPO), three 20-second “all-out” sprints at a resistance equivalent to 5% of baseline bodyweight with 2 minute recoveries at 10% PPO, and a 3-minute cool down a 10% PPO. Week 2 included 4 repeats and weeks 3 and 4 incorporated 5. Total training time per session ranged from 10 minutes during week 1 to ~15 minutes’ during weeks 3–4. The HIIT group completed a training program that has been widely used in the literature, i.e. 1 minute at 90% PPO with 1 minute recoveries at 10% PPO [15, 23]. As with SIT, all sessions began and ended with a 2-minute warm-up and 3-minute cool down. The first 2 weeks, participants completed 6 repeats and the final 2 weeks they performed 8. Total training time per session ranged from 16–20 minutes.

Sessions were completed at the same time of day within-participants (±1 hour). Dietary standardization was not strictly enforced; however, participants were instructed to refrain from eating ~2 hours before to avoid any gastrointestinal discomfort. Mean heart rates during the exercise sessions were used to determine the mean VO_2_ value during exercise based on the heart rate-VO_2_ relationship from an incremental maximal cycling test. Values for each session were calculated using the following equation, per McNeil and colleagues [13]:

$$Estimated\;exercise\;energy\;expenditure\;(ExEE)={VO}_{2}(L∙{kg}^{-1}∙{min}^{-1})\times Body\;weight\;(kg)\times Exercise\;duration\;(min∙{wk}^{-1})\times 5\;(kcal∙L^{-1}\;O_{2}$$ (1)

The four weeks of exercise were added together to yield a total exercise energy expenditure over the intervention.

We acknowledge, however, that the calculations above are not necessarily valid for intermittent non-steady state exercise. Therefore, we utilized two additional approaches to estimate ExEE. First, in our prior paper [17], we calculated ExEE using the equation of Dugas and colleagues for intermittent exercise [24]. Second, we calculated total work performed in each bout and converted work completed to energy expenditure using a typical value for gross efficiency [25]. Each exercise session’s energy expenditure was calculated individually and summed over the duration of the study. Comparisons between the methods (paired t-tests) revealed no significant differences in mean calculated ExEE and the associated predicted energy compensation (all p > 0.15).

### Outcome assessments

The primary outcome of interest in the current report was exercise energy compensation (%). Energy compensation was determined using the following equation described by others [13, 26, 27]:

$$Exercise\;energy\;compensation\;(\%)=\left(\frac{100}{ExEE}\right)\times [\left(\Delta FM\times 9500)+(\Delta FFM\times 1020\right)]+100$$ (2)

Where ExEE was the total energy expenditure of the exercise intervention, ΔFM and ΔFFM were the change in fat mass and fat-free mass from baseline, and 9500 and 1020 were the caloric densities per kilogram of fat mass and fat-free mass, respectively.

Another outcome examined was daily energy imbalance, calculated with the following equation [13, 26, 27]:

$$Energy\;imbalance\;(kcal∙d^{-1})=\left[1020\times (\frac{\Delta FFM}{\Delta t})\right]+\left[9500\times (\frac{\Delta FM}{\Delta t})\right]$$ (3)

Where ΔFM and ΔFFM were the change in fat mass and fat-free mass from baseline; 9500 and 1020 were the caloric densities per kilogram of fat mass and fat-free mass, respectively; and Δt was the length of the intervention in days.

Per the recent analysis by McNeil and colleagues, an energy compensation of 0% indicates that the change in body weight varied perfectly according to the exercise energy expenditure. A value of 100% indicates that no changes in body weight took place, despite the increased energy expenditure. A value above 100% would indicate weight gain, and a negative value would be indicative of weight loss [13].

Additional variables included in the present analysis were changes in body composition (Percent body fat (% BF), FFM, and FM; BodPod, Cosmed USA, Concord, CA); VO_2_max, via an incremental test on a cycle ergometer with breath-by-breath gas collection (ParvoMedics TrueOne 2400, Salt Lake City, UT, USA); NEPA (wrist-worn accelerometers placed on the non-dominant wrist for 7 days; GTX3+, Actigraph Corp., Pensacola, FL, USA); energy and macronutrient intake recorded over three consecutive days (Food Processor, ESHA Research, Salem, OR, USA); and RMR with a ventilated hood and metabolic system (ParvoMedics TrueOne 2400, Salt Lake City, UT, USA). Differences between pre- and post-testing (change scores) were calculated by subtracting baseline from post-testing values.

### Statistical analyses

Sample size for the original study was estimated based on prior research on interval training; additionally, based on an estimated β = 0.8, moderate effect size *f* = 0.25, and a correlation among repeated measures of 0.8 for resting metabolic rate, a total sample size of 21 participants was calculated using G*Power 3.1.9.2 [28].

In the original study, repeated measures ANOVA were conducted to examine the effects of time (pre/post), group (Control, SIT, HIIT), and their interaction (time*group). While the SIT and HIIT groups had training-induced changes in RMR and VO_2_max, they were not significantly different from each other. Independent-samples t-tests at baseline and post-testing showed no significant differences between the exercise groups. Specifically, no significant differences for exercise-induced changes in body composition, physical activity, exercise energy expenditure, and energy intake were observed between the SIT and HIIT groups during post-testing. Therefore, in order to increase power, the training groups were pooled in the present analysis. Following procedures previously used by McNeil and colleagues, we conducted a multivariable linear regression to examine the strength of the associations between energy compensation and changes in VO_2_max, energy intake, RMR, and NEPA, with baseline fat mass and VO_2_max as covariates [13]. Additionally, an independent samples *t*-test was conducted to determine differences in change scores between individuals who had energy compensation levels < 100% and those who had levels ≥ 100%. Data were analyzed using SPSS v. 23 (IBM Corp, Chicago, IL, USA), and statistical significance was accepted at *p* < 0.05.

## Results

A total of 24 participants underwent exercise training, with a further 6 serving as controls. The baseline characteristics of the individuals who performed training are reported in Table 1. There were no differences between the exercise groups at baseline when examined with independent *t*-tests.

**Table 1:** Characteristics of the exercise groups at baseline.

Variable	HIIT (n = 12)	SIT (n = 12)	Pooled	*P*-value*
Age (years)	30.5 ± 8.8	28.5 ± 6.1	29.5 ± 7.5	0.524
Weight (kg)	76.4 ± 16.2	81.2 ± 16.5	78.8 ± 16.1	0.482
BMI (kg∙m^-2^)	26.9 ± 3.6	28.4 ± 4.7	27.7 ± 4.2	0.377
Percent Body Fat (%)	25.7 ± 8.4	29.4 ± 10.7	27.5 ± 9.6	0.363
Fat mass (kg)	19.7 ± 7.7	24.7 ± 12.4	22.2 ± 10.4	0.243
Fat-free mass (kg)	56.3 ± 13.9	56.4 ± 9.8	56.3 ± 11.8	0.980
Maximal oxygen uptake (mL∙kg^-1^∙min^-1^)	31.4 ± 9.2	32.3 ± 7.1	31.8 ± 8.1	0.778
Resting metabolic rate (kcal∙d^-1^)	1670 ± 324	1789 ± 283	1730 ± 307	0.377
Energy intake (kcal∙d^-1^)	2371 ± 511	2515 ± 426	2443 ± 466	0.461

*independent samples *t*-test

**Fig 1:**
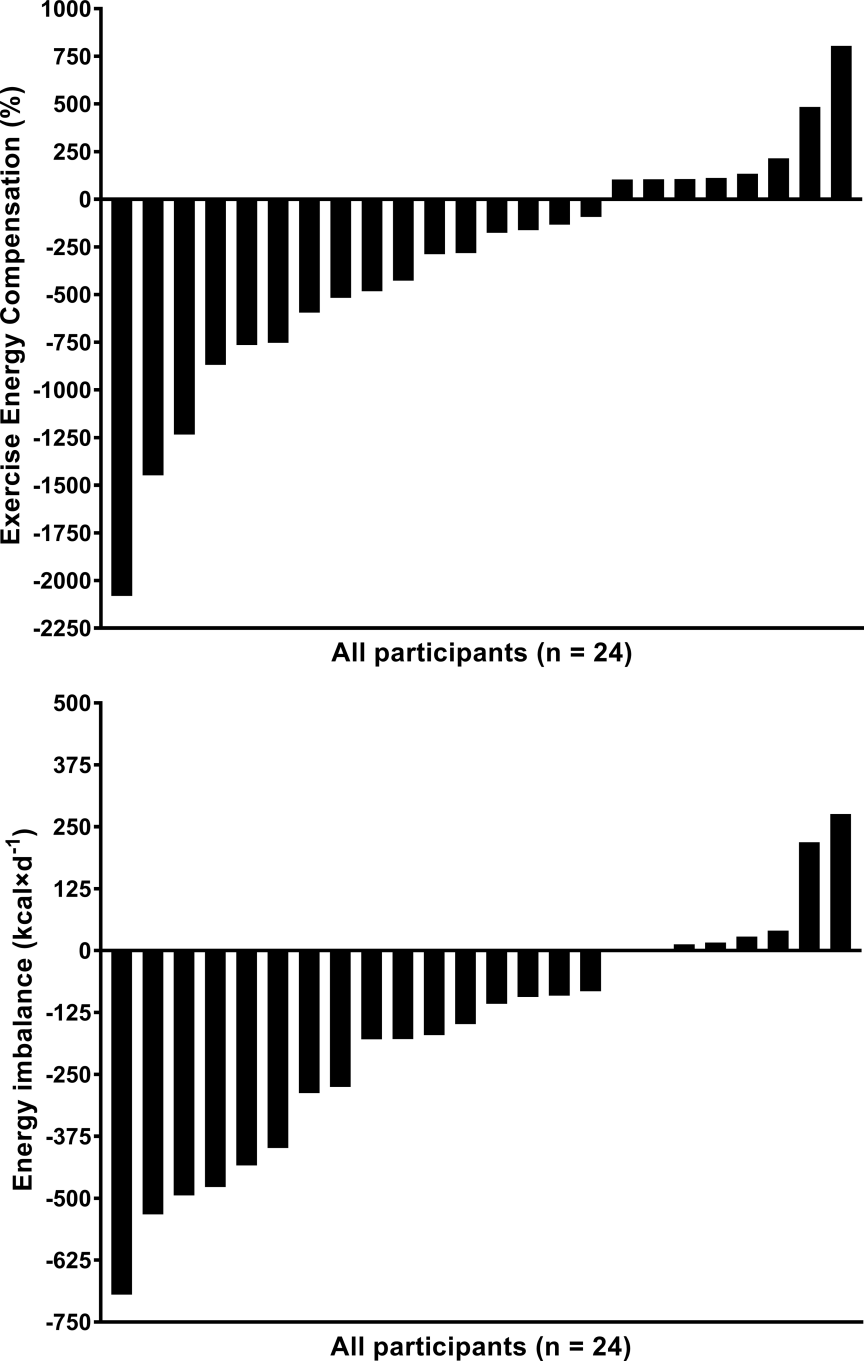
Distribution of individual exercise energy compensation (top) and distribution of individual daily energy imbalance (bottom).

Individual levels of compensation were highly variable. The results for individual values of energy compensation and daily energy imbalance are displayed in Fig 1. Energy compensation values ranged from -2081% to +805% (Mean ± SD: -346 ± 634%). Individual levels of energy imbalance also varied considerably, ranging from -695 to +276 kcal∙d^-1^ (-171 ± 239 kcal∙d^-1^). Two-thirds (n = 16) of the sample had compensation values < 100% with the remaining 8 participants having compensation levels ≥ 100%. The change scores for the variables of interest, based on compensation level, are shown in Table 2. Although both groups had minimal changes in body weight and BMI, participants who “over-compensated” (≥ 100%) gained fat-mass and lost fat-free mass, whereas those who did not overcompensate experienced the opposite trend (*p* < 0.0001 for FM and *p* = 0.008 for FFM, respectively). Additionally, individuals with lower levels (< 100%) of compensation had a 5-fold greater increase in VO_2_max (*p =* 0.008). Finally, daily energy imbalance was more negative in the individuals below 100% compensation, whereas those in the group that compensated ≥ 100% accrued a positive daily energy balance (*p* < 0.0001).

**Table 2:** Change scores by compensation level.

Variable	< 100% (n = 16)	≥ 100% (n = 8)	*P*-value
# Male/Female	6/10	4/4	0.558*
Weight (kg)	-0.26 ± 1.18	0.05 ± 1.09	0.285
BMI (kg∙m^-2^)	-0.06 ± 0.43	0.24 ± 0.43	0.329
Percent Body Fat (%)	-1.26 ± 1.16	-0.83 ± 1.29	0.439
Fat mass (kg)	-1.41 ± 0.81	0.33 ± 0.56	**<0.0001**
Fat-free mass (kg)	1.15 ± 1.25	-0.28 ± 0.76	**0.008**
Maximal oxygen uptake (mL∙kg^-1^∙min^-1^)	2.10 ± 2.05	0.41 ± 0.79	**0.008**
Resting metabolic rate (kcal∙d^-1^)	50.33 ± 42.10	35.61 ± 58.86	0.487
Energy intake (kcal∙d^-1^)	27.44 ± 81.43	22.13 ± 62.6	0.873
Physical activity (min∙d^-1^)	9.7 ± 40.1	-15.6 ± 32.1	0.135
Total ExEE (kcal)	1725 ± 525	1867 ± 261	0.547
Energy compensation (%)	-644 ± 548	251 ± 63	**0.0003**
Energy imbalance (kcal∙d^-1^)	-290 ± 191	67 ± 113	**<0.0001**

*Pearson Chi-Square test

Results of the linear regression are displayed in Table 3. In the first step, baseline fat mass and VO_2_max did not predict energy compensation (*F*_*2*,*23*_ = 1.17; *p* = 0.33). Adding ΔVO_2_max to the model in step 2 increased R^2^, but Model 2 also failed to significantly predict energy compensation (*F*_*3*,*23*_ = 2.97; *p* = 0.06). Adding ΔNEPA to the model in step 3 significantly increased R^2^, and Model 3 was found to significantly predict energy compensation (*F*_*4*,*23*_ = 7.95; *p* = 0.001). Thus, ΔVO_2_max (ΔR^2^ = 0.21, p = 0.024) and ΔNEPA (ΔR^2^ = 0.32, p = 0.001) both added significant variance to the final model, which predicted 63% of the variance in energy compensation.

**Table 3:** Predictors of energy compensation.

	*B ± SE*	*β*
**Model 1**		
Constant	-836.4 ± 913.1	---
Baseline FM	-5.8 ± 15.8	-0.10
Baseline VO_2_max	19.4 ± 20.3	0.25
R^2^ for Model 1	0.10	
**Model 2**		
Constant	-1079.6 ± 826.5	---
Baseline FM	1.7 ± 14.5	0.03
Baseline VO_2_max	29.4 ± 18.7	0.38
Change in VO_2_max	-156.6 ± 63.9	-0.47*
R^2^ for Model 2	0.31	
ΔR^2^ for Model 2	0.21*	
**Model 3**		
Constant	-979.8 ± 624.0	---
Baseline FM	-5.5 ± 11.1	-0.09
Baseline VO_2_max	34.4 ± 14.2	0.44*
Change in VO_2_max	-212.2 ± 50.1	-0.64**
Change in PA	-9.9 ± 2.5	-0.61**
R^2^ for Model 3	0.63	
ΔR^2^ for Model 3	0.32*	

*p < 0.025

**p < 0.0008 (n = 24)

## Discussion

The primary findings of the current study indicate that a greater change in non-exercise physical activity and VO_2_max predicts lower levels of energy compensation after interval training. These results suggest that exercise interventions which emphasize improving fitness and reducing sedentary time may result in reduced energy compensation–at least in the short-term, mandatory adherence environment of the present study.

Similar to McNeil and colleagues [13], we found that improvements in VO_2_max were associated with reductions in energy compensation. Those authors reported that their individuals with lower levels of compensation (< 100%) had greater (2-4x) increases in VO_2_max than those who had higher compensation levels (≥ 100%) [13]. While our sample size was much smaller, the present results revealed that those individuals with lower levels of compensation had an improvement in VO_2_max five times greater than those who had higher compensation levels. In contrast, McNeil reported a non-significant trend between energy compensation and change in objectively-measured NEPA [13]. Our results showed that individuals with lower levels of compensation accumulated ~25 more minutes of NEPA per day than those with higher levels of compensation, though this difference was not significant. In contrast to McNeil’s and our results, Rosenkilde and colleagues reported that no differences in ΔVO_2_max were observed between two doses of aerobic exercise, despite the moderate volume group having significantly lower compensation levels (-83% vs. +20%) [6]. Interestingly, despite the high-volume group having a 2-fold greater exercise energy expenditure per day, the moderate volume group had a more negative daily energy balance (-80 kcal). These authors also did not observe differences in NEPA between their intervention groups [6].

At this point, it would be prudent to consider the changes in light of the control group. It is not possible to calculate energy compensation for the control group, since their ExEE was zero. However, their daily energy imbalance was equal to 68 ± 45 kcal∙d^-1^, which is similar to what we calculated for the compensators. Thus, this raises an interesting point that perhaps the “compensatory” response we observed in the compensators may be within the normal variation. However, it has recently been posited that quantifying individual responses and changes is far more complicated than many would like to believe [29]. It is also worth considering that though the means for energy imbalance were similar, the standard deviation for the compensators was much higher than the control group.

The mechanism suggesting that greater changes in VO_2_max and NEPA are associated with lower exercise energy compensation is not clear. As early as the 1950s, it was suggested that activity levels and energy intake formed a J-shaped curve, which has also been replicated in recent research [30–32]. A comprehensive systematic review by Beaulieu and colleagues concluded that active individuals are more sensitive to the energy density of food, and may also have differences in body composition and sensitivity to appetite hormones implicated in energy balance (i.e. ghrelin, leptin, insulin) that may preclude them to be less likely to compensate in response to exercise training [31].

The primary limitation of the present study is the small sample size, which could increase the chances that our results are due to Type I error. Given the consistency of our results with those reported by McNeil [13], however, this is unlikely. Another limitation is our sample was young, healthy adults, and we lacked sufficient power to examine sex differences, though a meta-analysis previously found no influence of sex on the variation in energy compensation [10]. A third limitation related to the sample size is that we were unable to examine the effects of interval training intensity *per se* on energy compensation (i.e. a dose-response analysis). Another limitation is the use of self-reported food logs, which are likely to underreport energy intake. A limitation that our analysis shares with that of McNeil [13] is the relationship between VO_2_max and energy compensation may be driven by weight change. Neither these authors’ statistical procedures or our present analysis could include weight loss as a covariate due to it’s relationship with energy compensation; thus, the magnitude of change in VO_2_max could be a direct result of the amount of weight lost.

The nature of our calculation of energy expenditure from SIT and HIIT data is also worth considering. We acknowledge that without direct measures of oxygen consumption and/or muscle metabolism, it is difficult to precisely quantify EE. We also acknowledge that choosing an arbitrary value of 20% for gross efficiency may be contentious given the range of values reported in the literature for net or gross efficiency during high-intensity cycling (16–25%). Smith and Hill [33, 34] revealed differences in the aerobic contributions to Wingate testing based on the net or gross efficiency values used by themselves and other authors, though these differences appeared slight. Therefore, we acknowledge that our estimates of ExEE are only estimates, and that this in turn may have influences our derived values of energy compensation.

A final limitation is the duration of the intervention. Although we observed widespread variability of compensation, a meta-analytic review reported that negative compensation is more prevalent in short-duration studies, and attenuates as duration lengthens [10]. Thus, in future studies, a longer duration should be utilized.

In conclusion, we found that changes in VO_2_max and NEPA predicted energy compensation. Individuals who had greater improvements in VO_2_max and increased NEPA exhibited less compensation to exercise, and this was associated with greater loss of FM and retention of FFM without a change in body weight. This highlights the usefulness of targeting messages to the public that exercise can have beneficial impacts on factors other than weight loss. Future research should attempt to replicate these findings in a larger interval training cohort to examine dose-response effects, and include variables that are involved in energy compensation. This line of research could help determine causes of energy compensation and provide data to assist in the development of personalized and optimally effective exercise interventions.

## Supporting information

S1 Dataset (XLSX)
